# Computed Tomography and Magnetic Resonance Enterography: From Protocols to Diagnosis

**DOI:** 10.3390/diagnostics14222584

**Published:** 2024-11-18

**Authors:** Cesare Maino, Ilaria Mariani, Silvia Girolama Drago, Paolo Niccolò Franco, Teresa Paola Giandola, Francescamaria Donati, Piero Boraschi, Davide Ippolito

**Affiliations:** 1 Department of Diagnostic Radiology, IRCCS Fondazione San Gerardo dei Tintori, Via Pergolesi 33, 20900 Monza, Italy; i.mariani.dot@gmail.com (I.M.); sgd.drago@gmail.com (S.G.D.); francopaoloniccolo@gmail.com (P.N.F.); teresagiandola1990@gmail.com (T.P.G.); davide.ippolito@unimib.it (D.I.); 2 2nd Unit of Radiology, Department of Radiological Nuclear and Laboratory Medicine, Pisa University Hospital, Via Paradisa 2, 56124 Pisa, Italy; f.donati@med.unipi.it (F.D.); p.boraschi@do.med.unipi.it (P.B.); 3 School of Medicine, University of Milano Bicocca, Via Cadore 33, 20090 Monza, Italy

**Keywords:** magnetic resonance enterography, computed tomography enterography, inflammatory bowel disease, Crohn’s disease

## Abstract

Both Magnetic Resonance Enterography (MRE) and Computed Tomography Enterography (CTE) are crucial imaging modalities in the diagnosis and treatment of inflammatory bowel disease (IBD). CTE is often used in acute scenarios, such as when complications (such as abscesses, perforations, or bowel obstructions) are suspected. It can also help determine the degree and extent of pathological processes. Although CTE is rapid, generally accessible, and offers precise images that are useful in emergencies, it does expose patients to ionizing radiation. Nevertheless, MRE is very useful in assessing perianal illness and the small intestine, and it is frequently used in patients who need repeated follow-ups or are pregnant to minimize radiation exposure. Moreover, MRE can demonstrate oedema, fistulas, abscesses, and the thickening of the bowel wall. In addition, MRE offers superior soft tissue contrast resolution without ionizing radiation, which helps identify complications such as fistulas and abscesses. With their respective advantages and disadvantages, both approaches play essential roles in assessing IBD. The primary goal of this review is to provide an overview of the technical specifications, benefits, drawbacks, and imaging findings of CTE and MRE.

## 1. Introduction

With more patients visiting the radiology department with suspected inflammatory bowel disease and requiring diagnostic procedures, enterography imaging, such as Computed Tomography Enterography (CTE) and Magnetic Resonance Enterography (MRE), is becoming more common in daily practice.

In inflammatory bowel disorders (IBDs), cross-sectional imaging is a vital supplement to clinical and endoscopic examination [1,2,3,4,5,6]. This is primarily because endoscopic methods are unable to examine extra-intestinal disease extension (such as abscesses, sinus tracts, and fistulas) or transmural inflammation. Furthermore, as compared to endoscopy, the two most widely used methods now accessible in terms of tolerability are CTE and MRE. CTE and MRE help characterize disease phenotypes, activity, and response to therapy [7,8]. In addition, imaging allows for the examination of the jejunum and proximal ileum, which are inaccessible using conventional endoscopy [7].

In order to investigate the gastrointestinal system, the most widely used and recognized CTE and MRE protocols are outlined in this review, along with the key findings that should be kept in mind for routine clinical practice.

## 2. CTE or MRE?

According to the 2020 American College of Radiology (ACR) Appropriateness Criteria, the surveillance, acute exacerbation, and initial diagnosis of IBDs are all appropriate uses for CTE and MRE [9].

Because MRE does not involve ionizing radiation, it has become the noninvasive standard of reference for the pediatric population [10,11,12]. On the other hand, CTE is more accessible and quicker, and it can be used on patients with serious illnesses who are unable to attend an MRE examination, in order to obtain the proper diagnosis and course of therapy [13]. Furthermore, increased confidence and inter-reader agreement are produced by the inherent high spatial resolution and reproducibility of CTE [14].

The ECCO-ESGAR Consensus Guidelines do not provide precise indications regarding dose, pitch, and other technical parameters [15], which also depend on the vendors’ machinery. As a result, there is currently no clear consensus regarding which technical parameters guarantee the best diagnostic performance with the lowest possible radiation exposure. Up to 20% of IBD patients have a cumulative radiation exposure >50 mSv, which is the threshold for potentially dangerous radiation exposure and is equal to five abdomino-pelvic CT scans, according to Nguyen et al. [16]. Age and the duration of the condition generally increase the number of patients exposed to over-the-limit radiation. Several methods are employed to minimize radiation dose exposure, such as tube current (mA) modulation, lower tube potential modulation (kV), and a reduction in the number of CT phases [17,18].

CTE should be reserved for acute settings (e.g., bleeding and/or suspected acute bowel obstruction) [19,20] or in the case of claustrophobic patients. At the same time, MRE is usually preferred in stable patients for first-time assessment. Moreover, MRE performs better during follow-up than CTE, as it guarantees greater diagnostic confidence and reliability [21]. Furthermore, using endoscopy as a reference standard, MRE demonstrated a high accuracy in tracking therapeutic responses (an accuracy of 90% for ulcer healing, 83% for endoscopic remission, and 68% for anti-TNF response) [22]. Regretfully, MRE and CTE are not sensitive enough to identify pure mucosal lesions or to grade the disease [23,24]. However, MRE is able to differentiate inflammation and fibrosis [24,25]. Moreover, it can offer functional data from multiphasic and sequential static cine imaging, especially for scientific purposes [26]. Additionally, MRE enables high-resolution T2WI focused on the pelvis to categorize and rule out perianal fistulas [15,27] (Table 1 and Table 2).

## 3. Patient Preparation

### 3.1. Intraluminal Contrast Agents

The administration of oral contrast to distend the lumen is fundamental. Different preparations for CTE and MRE are available on the market. Firstly, all patients should fast for between 4 and 6 hours before the examination [2,11,12]. No consensus exists on how much contrast the patients should drink (different authors suggest using from as low as 450 mL up to 2000 mL). The Society of Abdominal Radiology-American Gastroenterological Association (SAR-AGA) consensus [7] introduced a weight-based approach, administering 20 mL/kg and up to 1350 mL of barium-containing contrast material (VoLumen).

The most commonly used oral agents in both CTE and MRE are PEG (polyethylene glycol), methylcellulose, and low-concentration barium (LCB), all of which have osmotic laxative properties [27,32].

CTE oral contrast media are distinguished between low attenuation (<25–30 HU) and iodine-containing oral agents. Neutral oral contrast agents are the most used luminal media (e.g., methylcellulose mixtures, PEG, lactulose, and milk [19]), as they better evaluate mucosal enhancement alterations [19,32,33,34]. In contrast, iodine-based positive contrast agents may help assess luminal and extraluminal complications (fistula, low-grade occlusion, abscesses, etc.) [34].

For MRE, the water-based biphasic contrasts lengthen both T1 and T2 times, resulting in decreased signal intensity on T1-weighted images (T1WI) and an increased signal on T2-weighted images (T2WI); these contrasts are considered ideal [35].

To provide adequate bowel distention, contrast administration can be performed per os (enterography) or through a nasogastric tube (enteroclysis). The crucial point is to obtain an adequate distention of the bowel lumen in 45–50 min. No routine bowel cleansing protocol is currently recommended [35,36].

Alternative administration routes can be considered in patients with intestinal-cutaneous stomia, through which the osmotic solution could be administered directly; additionally, after intestinal resection, the transit time reduces, and thus, the scanning procedure starts when contrast is visualized in the ileostomy bag or when watery bowel movements can be appreciated [19]. In younger patients (e.g., under six years of age) or those with cognitive impairments, enteroclysis MRI may be alternatively performed [37].

The usage of intraluminal contrast in the postoperative setting is controversial, as no data suggest it to be more sensitive for detecting anastomotic dehiscence [38]. However, if anastomotic leakage is suspected, positive oral and rectal contrast media could be considered to help the diagnostic process further [19].

### 3.2. Antiperistalsis Drugs

Peristaltic movements can impact disease location and extent evaluation, especially for MRE. To overcome this, antiperistaltic drugs can be administered, especially if no contraindications are present. According to Gandhi et al. [3], 81% of institutions use antiperistaltic medications before or during MRE, while 13% use it prior to CTE, with some variability in medication dose and administration method [3,11]. The consensus is that hypoperistaltic medications are required for MRE but may be avoided for CTE [11]. Other authors [39] underline that the suspension of peristalsis decreases bowel motion artifacts on post-contrast T1WI and minimizes luminal collapse, thus improving the images’ quality. The ECCO-ESGAR Guidelines suggest using spasmolytic agents for better image quality (preferably via endovenous administration), though their use may be avoided if contraindications occur [35,40].

Two main classes of drugs are recommended—butylscopolamine or glucagon [3,11]. A few authors [33,41] have suggested adding pro-kinetic drugs (e.g., metoclopramide) to spasmolytic drugs prior to MRE to enhance gastric emptying and provide better bowel distension. However, this approach’s validity has yet to be demonstrated [42].

### 3.3. Patients’ Positioning

The best way to position patients for scanning is still up for debate. However, the 2018 ECCO-ESGAR consensus guidelines [15] state that while prone positioning may result in better bowel distension, there is no proof that it improves diagnostic accuracy over supine positioning [2]. However, the prone position reduces motion artifacts, improving the signal-to-noise ratio (SNR) and contributing to separate bowel loops [11,26]. The choice of supine or prone position remains patient- and institution-dependent; however, prone positioning should be avoided in the presence of abdominal wounds or under general anesthesia [35].

## 4. Technical Considerations

### 4.1. CTE

To perform a CTE study, a 16-row CT scanner is a minimum requirement [19,43]; thin detector collimation and slice thickness are recommended (0.5–0.75 mm and 2–3 mm, respectively) [3,19,32,41]. These requisites also guarantee rapid image acquisition, minimizing motion artifacts [32]. However, the ECCO-ESGAR Joint Guidelines do not give a precise recommendation regarding technical parameters such as pitch, kV, and mAs; variations are contemplated depending on the machinery, though it is implicit that the maximal performance with the lowest dose possible should be achieved [15,32,40]. Multiplanar reconstructions (MPRs) in the coronal plane are mandatory [15,32], while the sagittal plane may be reserved for situations where diagnostic doubts arise; in the case of penetrating disease, orientated planes should be created [19] to evaluate pathologic processes and their relationship to surrounding structures.

As most authors and the 2018 ECCO-ESGAR Consensus Guidelines stated [15], iodinated contrast media is mandatory, though some concentration and scanning time variability may exist. The radiologist should adopt a non-ionic iodinated contrast media with ≥300 mg/mL of organic iodine (max. 60–70 g of organic iodine per patient) [19]. A slight discordance exists concerning the dosage, as some authors recur to a fixed amount of contrast (e.g., at least 120–150 cc [19]), while others recur to a weight-adjusted protocol.

The administration rate can vary from as low as 2 to 4–5 mL/s, though many authors [3,19,41] agree that it should be at least 3 mL/s, while higher rates (as of 4–6 mL/s [19]) are advised when better arterial phase enhancement is required (e.g., acutely ill patients with suspected active disease or possible endoluminal bleeding), in order to evaluate mucosal enhancement and wall stratification.

Scanning time should occur via the bolus-tracker method (BT), which involves the acquisition of a preliminary low-dose, single-slice scan at the diaphragmatic plane, over which an ROI (region of interest) is placed in the aortic lumen. When the density values inside the ROI reach the threshold (100–150 HU), the scanning time is started. While evaluating bowel segments, scanning occurs in the arterial phase (18 s from the start of BT—early arterial phase—or in the late arterial phase—at 25 s after BT) and the enteric phase (at 40–50 s). In the enteric phase, the small bowel enhancement peaks [19,43], thus allowing for the better evaluation of the transmural disease’s extent and location. According to many authors [18,34], to reduce the radiation dose, single post-contrast imaging in the enteric phase should be enough [18,34,41] unless GI bleeding or intraluminal hyperdense material is suspected.

The ECCO-ESGAR consensus guidelines suggest that image acquisition following intravenous contrast administration only occurs in the enteric or portal phase [40]. If iodinated contrast agents are contraindicated, fluoroscopic small bowel barium studies, MRE, or CT enteroclysis with positive enteral contrast can be alternatively performed [19].

### 4.2. MRE

The ECCO-ESGAR recommendations [40] state that MRE imaging can be performed using 1.5 T or 3 T scanners. Generally speaking, though, 1.5 T scanners are chosen due to their much greater availability and the reduction of band artifacts [26]. Despite having a more excellent SNR, 3 T scanners imply significantly more chemical shift effects, particularly on fat-saturated sequences at the air–water interface and on steady-state free-precession (SSFP) sequences [44]. This can result in false positive or false negative results. Furthermore, 3 T imaging shows increased susceptibility artifacts from surgical materials and intraluminal bowel gas [39,44]. Because surface phased-array coils provide a wider field of view and are more acceptable to patients, they are even advised to evaluate perianal diseases [26,27,45].

T2W images are the core of MRE in the axial and coronal planes, with a suggested slice thickness ≤ 4 mm [40,44]. Fat-saturated T1WI must be acquired in the coronal plane before and after gadolinium-based contrast injection. For contrast-based sequences, an intersection gap of 1.5 mm, with 25 images per slice, could provide adequate diagnostic quality [26]. Contrast-enhanced imaging should be performed dynamically and in the coronal plane, to cover the entire abdomen and pelvis in a single breath-hold [2]. The first acquisition should take place in the enteric phase (between 45 and 70 s) [2,27,39], and after at least two identical acquisitions, axial imaging of the abdomen and pelvis should be performed to adequately delineate anatomy and characterize extra-intestinal findings [2,39]. Subtraction images may increase the sensitivity of the detection of bowel wall hyperenhancement and fistulas [39]. The administration of 0.1 mg/kg paramagnetic contrast media, followed by a saline flush, is advised, usually with a flow rate of 2 mL/s [3,35,40,44].

As the ECCO-ESGAR Guidelines suggested, the scanning of the pelvis on T2WI or fat-saturated T1WI shall be included to assess for perianal disease if suspected [15,32].

Whenever paramagnetic contrast administration is contraindicated or not possible, DWI and cine-MRE could be considered a valid alternative, especially in pediatric patients [2,39,40]. Anupindi et al. [11] suggest using 3 to 5 *b* values, combining 0 and 1000 on 1.5 T scanners. The ECCO-ESGAR Guidelines suggest adding DWI to standard MRE protocols and combining them with T2WI to reduce the risk of false positive findings [32]. Apparent diffusion coefficient (ADC) maps could also help distinguish between acute and chronic inflammation [46].

Cinematic sequences are another instrument gaining consensus in the IBD imaging protocol; cine-MRE may prove very useful in distinguishing actual stenotic tracts from collapsed bowel loops [11,34,47]. They consist of balanced steady-state-free precession images (BSSFPs) [26,39]. The acquisition should be performed during free breathing and in the coronal plane [26]. Though reduced bowel motility correlates with the disease’s activity, cine-MRE is not routinely performed according to the ECCO-ESGAR Guidelines [15,40].

## 5. Imaging Features: What to Focus on

**Figure 1:**
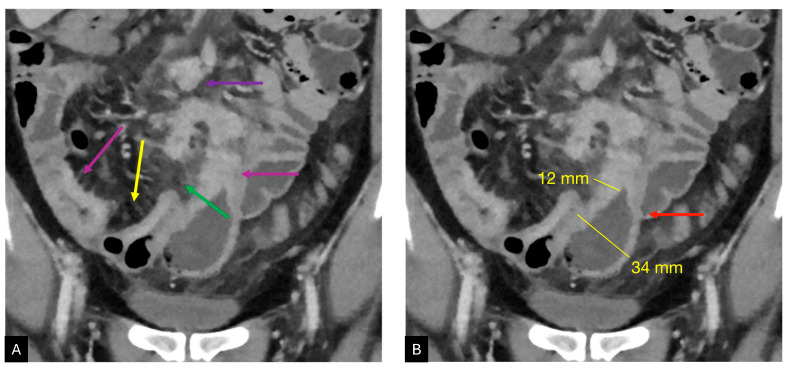
A 52-year-old male underwent CTE for bowel evaluation with a known history of Crohn’s disease. The patients reported multiple episodes of diarrhea, with increased laboratory inflammatory indexes. CT coronal multiplanar reconstruction on the portal venous phase is reported in (**A**,**B**). The most critical findings in (**A**) are diffuse and multiple small bowel wall thickening (red arrows) associated with comb sing (yellow arrow), peri-visceral oedema (green arrow), and enlarged nodes (purple arrow). In (**B**), it is possible to detect a significant thickening of a small bowel loop (red arrow), with homogeneous contrast enhancement, associated with a slight dilation of the upstream bowel portion.

**Figure 2:**
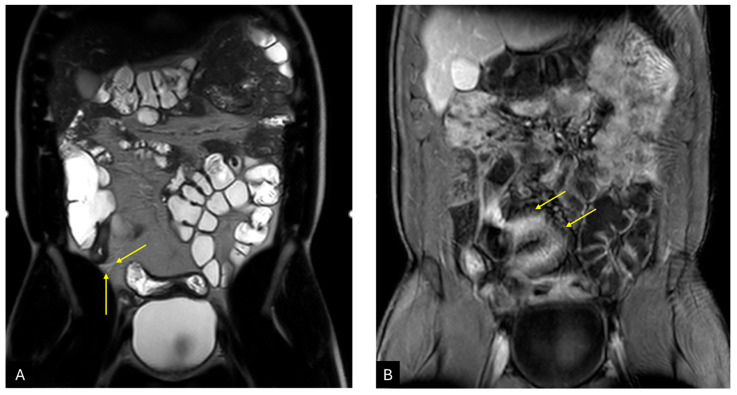
A 44-year-old male suspected of Crohn’s disease underwent MRE for the evaluation of bowel loops. The patients reported multiple episodes of constipation and diarrhea, with laboratory inflammatory indexes in range. Coronal T2WI (**A**) and T1WI (**B**) after contrast media administration. The most important finding is the thickening of the terminal ileum (**A**—yellow arrows), characterized by increased contrast enhancement (**B**—yellow arrow). Notably, the enhancement pattern is known as trilaminar, considering the lack of enhancement of the submucosal layer. The reported findings align with Crohn’s disease in the activity phase.

Based primarily on wall thickness and increased intravenous contrast uptake, CTE and MRE are used to assess the severity and activity of the disease [14] (Figure 1 and Figure 2). Both methods exhibit a good sensitivity and a similar specificity when CTE and MRE are directly compared for identifying different small intestinal lesions [31,48,49]. To create clinically useful radiology reports, radiologists aim to identify the major imaging features associated with small bowel and characterize these findings. In a recent article by the Society of Abdominal Radiology Crohn’s Disease–Focused Panel [47], many of the CTE and MRE imaging findings are illustrated; they also recommended standardized radiology report statements to summarize the findings of small bowel Crohn’s disease at CTE and MRE. The consensus recommendations included CTE and MRE bowel wall findings associated with Crohn’s disease, findings with penetrating subtype, and changes in the mesentery.

**Figure 3:**
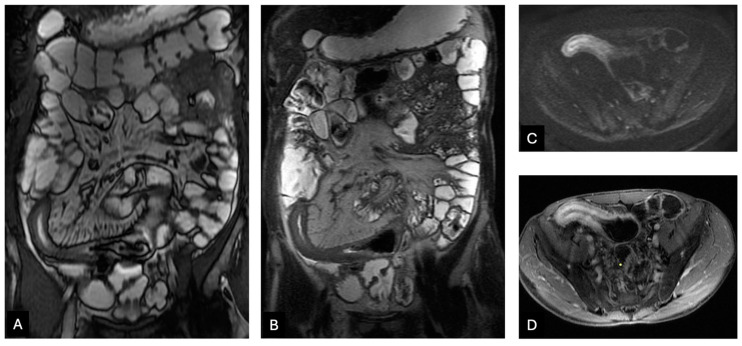
A 49-year-old man with Crohn’s disease involving the neo-terminal ileum. Coronal FIESTA (**A**) and SSFSE T2WI (**B**) show a thickened neo-terminal ileum with an increased T2 signal. Axial DWI (*b* = 1000) (**C**) image at the same level reveals increased intramural signal, indicating restricted diffusion, and the post-contrast axial T1WI (**D**) demonstrates intensely, layered pattern enhancement (mural stratification), consistent with active inflammation.

Bowel wall imaging findings typically include segmental mural hyperenhancement (Figure 3), which is defined as increased mural attenuation at CTE or increased mural signal intensity at MRE on contrast-enhanced images in a non-contracted small bowel segment compared with that of regular small bowel segments [14,50,51,52]. The appearance of mural hyperenhancement can be asymmetric, stratified, or homogeneous. Asymmetric mural hyperenhancement usually involves the mesenteric border of a small bowel loop more than the antimesenteric margin. Stratified mural hyperenhancement is characterized by the hyperenhancement of the inner or both the inner and outer aspects of the bowel wall. In contrast, the homogeneous symmetric mural hyperenhancement is transmural and uniformly involves the entire bowel wall.

Bowel wall thickening is another imaging feature that should be assessed and measured in a bowel segment that is adequately distended; it can be classified as mild (3–5 mm), moderate (>5–9 mm), or severe (≥10 mm) [53,54]. Wall thickening should be measured at the thickest portion of the most distended segment or in correspondence with the most inflamed bowel.

The high signal intensity of the intestinal wall on fat-suppressed T2WI or DWI is an imaging characteristic of mural oedema; this imaging feature cannot be as adequately assessed with CTE due to decreased contrast resolution compared with that of MRE [31]. Active inflammation associated with Crohn’s disease has been demonstrated to limit diffusion in the intestinal wall, even though this is not a distinctive symptom. On high b-value diffusion-weighted images, bowel segments with restricted diffusion exhibit a significant signal intensity [55,56]. However, whenever the bowel is not adequately distended (especially in the jejunum), bowel segments may demonstrate spurious hyperintensity on DWI, and radiologists should promptly recognize this.

**Figure 4:**
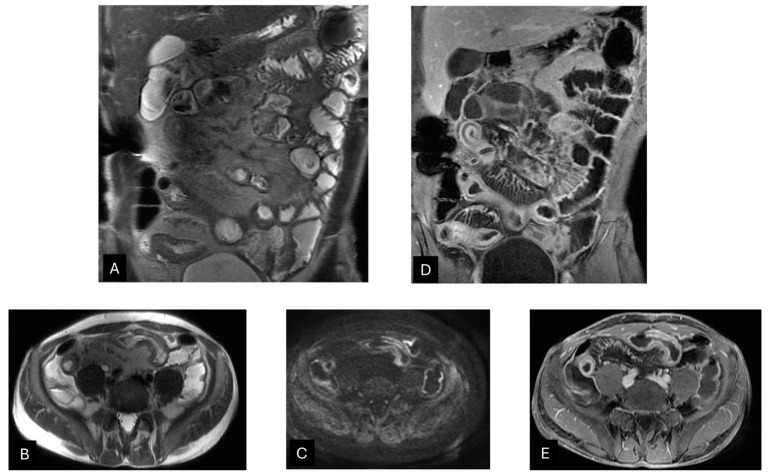
A 56-year-old man suffering from Crohn’s disease affecting the mid-distal section of the ileum with stenotic/substenotic and ecstatic features. Coronal (**A**) and axial (**B**) T2WI SSFSE images show multiple wall thickenings with a high T2 signal at the level of the ilium. The axial DWI (*b* = 1000) (**C**) image reveals increased intramural signal intensity, indicating restricted diffusion of the multiple thickened tracts. Coronal (**D**) and axial (**E**) post-contrast T1WI demonstrate intense wall structure multilayer enhancement.

The presence of a bowel stricture is a crucial imaging feature in Crohn’s disease; it is defined as the luminal narrowing (a luminal diameter reduction of at least 50% in comparison with that of a standard adjacent loop) of a bowel segment with upstream bowel segment dilation (≥3 cm) [57,58] (Figure 4).

A penetrating complication that develops adjacent to a stricture may decompress the upstream small bowel, resulting in no upstream dilation. Strictures can be present with or without active inflammation. In the presence of a stricture, the location and length of the stricture should be described together with signs of concurrent inflammation or upstream dilation. The association of a stricture with an enteric anastomosis should also be mentioned. In addition, there is growing evidence that stricture formation can be associated with penetrating disease in the small bowel [59,60]. Therefore, if a bowel stricture with active inflammation is present, assessing the presence of a penetrating disease such as a fistula is essential. Conversely, if a fistula or inflammatory mass is present, it is crucial to identify an adjacent strictured bowel loop that is typically associated with active inflammation.

On both CTE and MRE, ulcerations are imaging characteristics that are difficult to detect. They manifest as a tear in the intestinal wall’s endoluminal surface, allowing intraluminal fluid to penetrate the wall [53,61,62]. An ulcer is, by definition, a parietal defect contained within the intestinal wall and does not spread outside the serosa. Imaging findings of penetrating Crohn’s Disease are represented by simple and complex fistula, sinus tract, inflammatory mass, abscess, and rarely by free perforation. All these imaging features are well appreciable both on CTE and MRE.

A blind-ending tract extending beyond the bowel wall serosa but not reaching adjacent organs or tissues is defined as a sinus tract, whereas a simple fistula is characterized by a single extra-intestinal tract that connects the bowel lumen to another epithelial surface; a simple fistula may or may not contain fluid and usually occurs in the setting of a stricture with active inflammation [59,60,63]. Fistulas can be entero-enteric, entero-colic, entero-cutaneous, entero-vesical, or recto-vaginal [64]. Conversely, the presence of more than one fistulous tract defines a complex fistula; it is usually represented by multiple entero-enteric or entero-colic fistulas extending into the adjacent mesentery, with a typical asterisk appearance at MRE or CTE imaging [65].

An inflammatory mass (dense mesenteric inflammation without a well-defined fluid component or wall, which occurs adjacent to an inflamed bowel wall) or an abscess (well-delimited fluid collection) may also be present in this setting. An inflammatory mass is usually composed of ill-defined soft tissue attenuation on CTE or variable signal intensity on MRE images mixed with fat; on the other hand, an abscess shows a fluid component with typical rim enhancement on contrast-enhanced CTE or MRE due to the presence of a well-formed wall (with or without internal gas). Abscesses generally have restricted diffusion with high signal intensity on high *b*-value diffusion-weighted images.

At last, mesenteric features associated with small bowel Crohn’s disease are represented by peri-enteric oedema or inflammation (increased attenuation on CTE or increased signal intensity on T2WI MRE in the mesenteric fat adjacent to the diseased bowel loops), by the “comb sign” (engorged vasa recta as enlarged blood vessels that supply and drain an inflamed bowel loop), by fibrofatty proliferation (hypertrophy of the mesenteric fat adjacent to diseased bowel segments showing slightly increased attenuation on CTE and slightly decreased signal intensity on T1WI MRE compared with that of normal fat), by mesenteric venous thrombosis or occlusion, and by mesenteric lymphadenopathies.

Radiologists must recognize and accurately characterize small bowel Crohn’s disease imaging findings immediately. Several templates have been put forth to assist radiologists—especially those lacking expertise—in reporting all imaging data pertinent to patients with Crohn’s disease. Multidisciplinary meetings are also perfect for providing clinicians access to all relevant data.

## 6. Disease Grading

Research into scoring systems combining radiologic and clinical features capable of correlation with histologic data is currently a primary field of study. The advantage of severity scoring systems is that they integrate imaging findings systematically and reproducibly [4], even though they cannot reflect inflammatory severity variation over a bowel loop. There are few cross-sectional indexes for Crohn’s disease, all limited to MRE, among which the most used is the MR Index of Activity (MaRIA), which has a high sensitivity, specificity, and diagnostic accuracy for ulcerative lesions (sensitivity: 78.3%; specificity: 98%) [22]. The MaRIA score was first introduced in 2011 by Rimola et al. [53]; it is calculated separately for each bowel segment, and the global score is the sum of each segment’s score. Many authors highlighted the high correlation between the MaRIA score and endoscopy [66,67], further underlying one of the main advantages of MRE towards endoscopy. In addition, the MaRIA score allows for a per-segment analysis comparable to endoscopic evaluation and is extremely useful considering the skip-lesion pattern of Crohn’s disease [53]. The MaRIA score is calculated using the following formula: 1.5 × wall thickness + 0.02 × RCE [relative contrast enhancement] + 5 × oedema + 10 × ulceration. One of the main disadvantages of the MaRIA score is that its calculation is relatively complex, as it requires a separate assessment of the bowel segments, which delineates an ROI (region of interest) to calculate the RCE (relative contrast enhancement). Additionally, not all authors agree that MRE’s indexes are equivalent to endoscopic findings, especially when compared to capsule endoscopy [68].

However, the MaRIA score is not the only MRE score recognized by the radiological community; a few others (e.g., the London Score and the Nancy Score) are applicable, and despite their differences, they all correlate well with endoscopy and can change the treatment approach (surgical vs. medical).

The Clermont Score (or DWI-MaRIA scoring system) can also be applied whenever contrast injection cannot be performed. Buisson et al. [69] highlighted that DWI correlated with disease activity. Sensitivity, specificity, and positive and negative predictive values were found to be as high as 100%, 92.9%, 94.4%, and 100%, respectively. Quantitative analysis was performed by designing ROIs on the ADC maps in the axial plane, placed on the most significant area covering the bowel wall. Though very promising, the Clermont Score did not overstep the MaRIA score, mainly because of the variability of ADC map calculation depending on MR machines’ software, which results in scarce comparability between different vendors and institutions. Hordonneau et al. confirmed Buisson’s results; scores > 8.4 are highly predictive of ileal CD activity, while a Clermont score ≥ 12.5 is highly predictive of severe ileal CD; they also found a high interobserver agreement, with accuracy rates as high as 99.2% per segment [70].

Even if the Clermont and the MaRIA indexes require complex calculations, the Nancy Score overcomes this inconvenience, as it implies assigning 0 to 1 points per indicator for each bowel segment (distinguishing among rectum, sigmoid colon, descending colon, transverse colon, ascending colon, and terminal ileum). A segmental Nancy score is the sum of the numerical values obtained for the six radiological signs for a single segment [71]. The Nancy score focuses on mucosal healing as the primary goal (while other scores are more oriented on the evaluation of disease severity) and, as well as the Clermont score, suffers from the lack of standardization and post-processing variability in the ADC’s acquisition method. Moreover, as Choi et al. [72] pointed out, DWI cannot substitute contrast administration, as its application alone may result in a high rate of false positive results, and its interpretation should always correlate with DCE findings to assess actual inflammatory bowel disease.

Aiming to assess response to treatment, all of the mentioned radiologic ratings have undergone validation. The only metric available to evaluate the activity of the disease is the London score, which was first published by Steward et al. [73] in 2012 and was validated with reference to surgical specimens of resected ileal segments. Since there is yet to be a radiologic score that is frequently used in clinical practice, more research is required to validate these findings (Table 3).

## 7. Cross-Sectional Imaging Limitations

The integration of enterography imaging and endoscopy is still under assessment, as both techniques retain specific strengths and weaknesses; MRE and CTE changed the scenario, allowing for a more conservative approach than that required by endoscopy. However, though they can depict extraluminal complications better than colonoscopy alone [41,68,74], we still cannot entirely rely on imaging for the diagnosis and follow-up of IBDs. Different authors established that both CTE and MRE guarantee relatively high performance in the detection of IBD [25], with CTE achieving a sensitivity, specificity, and accuracy as high as 98%, 95%, and 97%, respectively, when compared to ileocolonoscopy. Similar results have also been obtained regarding MRE, which some authors have proven to have a reported sensibility of between 86% and 95% and a specificity between 84% and 92% in detecting bowel wall abnormalities compared to ileocolonoscopy and histology [75]. A systematic review and meta-analysis by Yung et al. [76] comparing the performance of MRE vs. ileocolonoscopy in detecting postoperative complications in Crohn’s disease found that the pooled sensitivity of MR enterography for the detection of endoscopic recurrence was 97.3%, with a pooled specificity of 83.7% and an AUC of 0.98. Unfortunately, there is currently insufficient research on this subject, so imaging cannot consistently replace endoscopy. Of course, because endoscopy is a more invasive technique with a relatively significant risk of periprocedural complications, patients typically prefer MRE or CTE over it. Additionally, imaging makes it possible to identify jejunal and ileal problems that endoscopy typically misses. On the other hand, endoscopy makes it possible to obtain a tissue sample for histology, which is essential for diagnosis and follow-up (particularly in patients receiving immunomodulator treatment) and cannot be obtained otherwise.

## 8. Conclusions

Because of their increased repeatability, low cost, and widespread availability, CTE and MRE have gained an essential role in the diagnosis, staging, and follow-up of patients with IBDs in recent decades. Both approaches can be regularly used in clinical practice and have benefits and drawbacks. Given their core responsibilities, radiologists should be familiar with the most crucial advice and potential problems. Furthermore, imaging findings on both techniques should be well understood to aid clinicians in managing patients.

## Figures and Tables

**Table 1:** Main advantages and disadvantages of CTE and MRE imaging.

CTE		MRE	
Advantages	Disadvantages	Advantages	Disadvantages
- Fast and widely available - Easy to perform - Assessment of extra-intestinal organs - Better interobserver agreement - High image quality	- Radiation dose exposure - Contraindicated if CKD/RF and severe contrast anaphylaxis	- No ionizing radiation - High tissue contrast - Informative even without contrast media administration - Both anatomic and functional study	- Time-consuming - Technically more difficult - Not widely available - More expensive - Motion artifacts - Variable image quality

CTE: Computed Tomography Enterography; MRE: Magnetic Resonance Enterography; CKD: Chronic Kidney Disease; RF: Renal Failure; GI: gastrointestinal.

**Table 2:** Reported sensitivity and specificity of CTE and MRE.

Authors	CTE		MRE	
	Sensitivity (%)	Specificity (%)	Sensitivity (%)	Specificity (%)
Duigenan et al. [28]	n/a	n/a	81–91	67–89
Liu et al. [29]	87 (95% CI, 78–92%)	91 (95% CI, 84–95%)	86 (95% CI, 79–91%)	93 (95% CI, 84–97%)
Greenup et al. [23]	67–95	70–90	66–100	72–100
Horsthuis et al. [30]	84.3 per patient analysis67.4 per segment analysis	95.1 per patient analysis90.2 per segment analysis	93 per patient analysis70.4 per segment analysis	92.6 per patient analysis94 per segment analysis
Gomollòn et al. [1]	84 per patient analysis	n/a	93 per patient analysis	n/a
Fiorino et al. [31]	Strictures’ detection: 85Per-segment analysis: 81Rectal disease: 81	Strictures’ detection: 51Per-segment analysis: 81Rectal disease: 50.9	Strictures’ detection: 92Per-segment analysis: 93Rectal disease: 72	Strictures’ detection: 90Per-segment analysis: 72Rectal disease: 100
Maaser et al. [15]	92	100	89	94

CTE: Computed Tomography Enterography; MRE: Magnetic Resonance Enterography; n/a: not applicable; CI: confidence interval.

**Table 3:** Summary of the currently validated MRE scores for the evaluation of disease activity.

	MaRIA	Simplified MaRIA	London	Nancy	Clermont
Fasting	Yes	Yes	Yes	No	Yes
Bowel preparation	Yes	Yes	Yes	No	No
Oral contrast administration	Yes	Yes	Yes	Yes	Yes
Gadolinium-based i.v.	Yes	No	Yes	Yes	No
Motility assessment	No	No	No	No	Yes
Bowel wall thickness	Yes	Yes	Yes	Yes	Yes
Wall enhancement	Yes	No	Yes	No	No
Edema	Yes	Yes	Yes	Yes	Yes
Ulceration	Yes	Yes	No	Yes	Yes
Mesenteric node enlargement (>1 cm)	Yes	No	Yes	No	Yes
Cut-off values for endoscopy correlation	≥7 for active disease ≥11 for severe ulcerative disease	>1 for active disease >2 for severe lesions	≥4.1 for the presence of histopathological acute inflammation	Mucosal healing: ≤6 total Nancy score ≤2 segmental Nancy score	>8.4 for ileal activity ≥12.5 for severe ileal disease

MaRIA: Magnetic Resonance Index of Activity; PEG: polyethylene glycol.
